# Magnetogel Nanospheres Composed of Cisplatin-Loaded Alginate/B-Cyclodextrin as Controlled Release Drug Delivery

**DOI:** 10.15171/apb.2019.065

**Published:** 2019-10-24

**Authors:** Ali Darini, Touba Eslaminejad, Seyed Noroddin Nematollahi Mahani, Mehdi Ansari

**Affiliations:** ^1^Department of Nanotechnology, Pharmaceutical Sciences Branch, Islamic Azad University (IAUPS), Tehran, Iran.; ^2^Pharmaceutics Research Center, Institute of Neuropharmacology, Kerman University of Medical Sciences, Kerman, Iran.; ^3^Department of Anatomy, Afzalipour School of Medicine, Kerman University of Medical Sciences, Kerman, Iran.; ^4^Neuroscience Research Center, Institute of Neuropharmacology, Kerman University of Medical Sciences, Kerman, Iran.

**Keywords:** Alginate, Beta-cyclodextrin, Cancer, Cisplatin, Controlled release, Drug delivery systems, Magnetic nanoparticles

## Abstract

***Purpose:*** The main aim of the present study was to design, fabrication and physicochemical characteristics of the magnetogel nanospheres as carriers for Cisplatin in the in vitro environment.

***Methods:*** Magnetic nanospheres were synthesized by using a chemical co-precipitation method and coated by sodium alginate through double emulsion method. Then cisplatin was encapsulated into β-cyclodextrin -sodium alginate grafted magnetic nanospheres. The physicochemical properties of the sodium alginate grafted magnetic nanospheres were characterized by using FTIR, particle size analyzing, vibrating sample magnetometry, thermogravimetric and SEM analysis. Also the drug entrapment efficiency, content and in vitro release profile were investigated.

***Results:*** Size distribution results revealed that the particles size was distributed in the range of 50± nm. Also morphological properties showed that particles are separated and spherical with the grafted layers of the polymer. The release profile data were in the acceptable range compared to the blank (cisplatin solution).

***Conclusion:*** It could be concluded that the sodium alginate grafted magnetic nanospheres could act as a slow and controlled release system to deliver cisplatin.

## Introduction

Cancer incidence and mortality rates increase year by year, making it a serious threat to human health. Conventional therapy methods cannot completely eliminate the cancer cell. In this part, chemotherapy is efficient adjuvant approaches to removing residual cancer cells and preventing tumor recurrence and metastasis, but their application is limited because of their serious side effects. Thereof, controlled drug delivery systems that allow releasing of drugs just at the desired site (cancerous cells) for extended periods time are needed. Furthermore, the significant effects of the targeted drug delivery lie to minimize toxic effects. Currently, cis-diammine dichloroplatinum (CP), as the first platinum-containing a chemotherapeutic agent is widely used in the treatment of solid tumors. Cell division is arrested by CP to prevent tumor growth and induces programmed cell death. Unfortunately, CP displayed serious side effects such as nephrotoxicity, ototoxicity, neurotoxicity, nausea, and vomiting because of systemic administration, which limits its clinical utilization.^1,2^ Thereof, to avoid these side effects CP is loaded on the nanocarriers to enhance permeability retention effects. Nanotechnology plays a unique role in cancer diagnosis and therapy^3^ and provide controlled drug release with increased tumor selectivity and decreased toxicity.^4,5^ Applying magnetic nanoparticles in the presence of an external magnetic field focus on the target tissue has already been demonstrated as a promising approach for the specific delivery of therapeutic agents.^6,7^ Iron oxide nanoparticles are widely used as contrast agents and drug carriers in preclinical and clinical settings.^8^

Polymeric delivery systems have the potential to maintain therapeutic levels of a drug, to reduce side effects and to facilitate the delivery of drugs with short *in vivo* half-lives.^9^ Because of several unique properties of alginate, it was allowed to use for the entrapment the delivery of a variety of biological agents.^10^ Alginate is non-toxic, biodegradable, low in cost, readily available, and mucoadhesive, biocompatible, and non-immunogenic substance.^11,12^ It is an anionic polymer and composed of two types of uronic acid monomers distributed as blocks of 1→4 linked α-L-guluronic acid (G) or β-D-mannuronic acid (M), as well as heteropolymeric mixed sequences (G-M, usually alternating). Cyclodextrins (CDs), a family of macrocyclic oligosaccharides linked by α-1, 4 glycosidic bonds, have been widely studied in various ﬁelds. Among them, α-, β-, and γ-CDs are the most common members, which are composed of 6, 7, and 8 glucose units, consecutively.^13,14^ The hydrophobic cavity of CDs gives them inclusion capacity with a diversity of compounds from small molecules, ions, proteins, and oligonucleotides. To further improve the pharmaceutical features of native CDs, chemically modified CDs have been synthesized.^15-17^ Therefore, in the present work CP was conjugated with β-CD and then loaded on the Fe_3_O_4_/alginate nanospheres. Physicochemical characteristics of the new synthesis particles were determined by using FT-IR, TGA, SEM, and TEM. Finally, drug entrapment efficiency (%), drug content (% w/w) and the *in vitro* release profile of CP-magnetic nanospheres were determined.

## Materials and Methods

### Materials

CP hydrochloride was purchased from Sobhan Oncology Co., Tehran, Iran. β-CD was purchased from Walker Chemie, Munich, Germany, o-Phenylenediamine and potassium dihydrogen orthophosphate, dimethylformamide (DMF), ammonium hydroxide (25%) and sodium hydroxide flakes were purchased from Merck, Darmstat, Germany. FeCl_2_- 4H_2_O, FeCl_3_-6H_2_O, and sodium alginate were purchased from Sigma-Aldrich, Louis, MO, USA, and used without further purification except when mentioned specifically. Dulbecco’s modified eagle’s medium F12 (DMEM), fetal bovine serum (FBS), penicillin–streptomycin (100 μg/mL), phosphate-buffered saline (PBS), MTT (3-(4, 5-dimethylthiazol-2-yl)-2, 5-diphenyltetrazolium bromide), and dimethyl sulphoxide (DMSO) were purchased from the Sigma Aldrich Company, Mo, USA.

### Methods

#### Preparation of Fe_3_O_4_nanoparticle

Fe_3_O_4_ nanoparticles were prepared by using co-precipitated method with some modification.^18^ Briefly, 35 mL of 1 mol/L FeCl_2_ and 1.5 mol/L FeCl_3_ suspensions were added into a three-necked flask to make the molar ratio of Fe^2+^ and Fe^3+^ maintaining 1:1.5, and was stirred under nitrogen atmospheres. Then ammonia (25% w/w) was added to regulate the pH value to 11 at 60°C and the dark suspension was incubated on vigorous stirring at 80°C for 1 h. Finally, the Fe_3_O_4_ black precipitate was allowed to cool to room temperature (25°C) and the separated by centrifugation at 6000 rpm for 20 min. The dark precipitate was lyophilized and stored in desiccators for later experiments.^19^

#### Preparation of CP encapsulated superparamagnetic alginate/β-cyclodextrin nanospheres (Fe_3_O_4_/A/β-CD/CP)

Firstly a stock solution of 20 mg of cisplatin was prepared in DMF and then diluted with 4.2 mL of deionized water (DDW), sonicated for 12 min to have a uniform suspension and kept on the dark conditions (wrap in the Aluminum foil). β-CD (76 mg) was dissolved in 20 mL of DDW. Then CP suspension was added drop-wise to the β-CD solution and sonicated at 22°C for 10 min to encapsulate CP in the β-CD. CP encapsulated β-CD colloids were drop-wise to the sodium alginate colloid (160 mg in the DDW) under stirring (A/β-CD/CP). Fe_3_O_4_ (48 mg) was suspended in a ratio of 2:1 (V/V) of ethanol: DDW in an ultrasonic bath at 22°C for 10 min. Finally, A/β-CD/CP composite was drop-wise into Fe_3_O_4_ suspension under agitation. The suspension was vibrated in an ultrasonic bath and stirred vigorously by a homogenizer simultaneously at room temperature for 30 min. Then, 14 mL of 0.01 M CaCl_2_ solution as cross-linker and 3.2 mL of 0.12 M NaHCO_3_ solution were poured into the mixture and stirred for 2 h. The suspension was separated by a permanent magnet and washed with ethanol and distilled water and then freeze- dried for later experiments.^19^

#### Characterization of the fabricated particles

FT-IR spectra from β-CD, CP, sodium alginate, Fe_3_O_4_, Fe_3_O_4_/A/β-CD and Fe_3_O_4_/A/β-CD/CP were recorded by a Fourier transforms infrared spectrophotometer (Tensor 27, Bruker Co.). Particle size, poly dispersity index, and zeta potential were measured using dynamic light scattering with a Nano-S90 Zeta Sizer (Malvern Instruments, Worcestershire, UK). All experiments were carried out at 25°C and repeated three times. The morphological studies of Fe_3_O_4_/A/β-CD and Fe_3_O_4_/A/β-CD/CP were observed by transmission electron microscopy (TEM, CM-10 PHILIPS, 80 kV) and scanning electron microscopy (FE-SEM, MIRA 3 XMU, Tescan USA Inc.). The magnetization curves of Fe_3_O_4_, Fe_3_O_4_/A/β-CD, and Fe_3_O_4_/A/β-CD/CP were measured with a vibrating sample magnetometer (VSM-7300, Meghnatis Kavir Co., Kashan Iran). The differential thermal analysis (DTA) coupled with thermogravimetric (TG) analysis of Fe_3_O_4_/A/β-CD and Fe_3_O_4_/A/β-CD/CP were observed by a TG-DTA apparatus (NETZSCH-GERÄTEBAU GMBH-STA 409 PC LUXX, Kerman, Iran) by heating the samples from 25 to 900°C at a heating rate of 10^o^C per min under an N_2_ atmosphere.

#### Drug loading measurements

One milliliter of Fe_3_O_4_ /A/β-CD/CP suspension was reached to 10 mL by DDW and then the free drug was separated by centrifugation at 6000 rpm for 15 min. Then after the free drug was measured according ortho-phenylenediamine method.^1^ Briefly, one ml of the supernatant was transferred to a 10 mL tube with a screw cap, and the measurement of the absorbance of the CP was done at 705 nm. Finally, the amount of the drug that was loaded on the particles was calculated by using the following formula:

Drug loaded (%)= total amount of drug / amount of the drug that was recovered from the supernatant.

#### In vitro release profile of CP

Dynamic dialysis method was used to investigate the *in vitro* release of CP. Briefly, Fe_3_O_4_/A/β-CD/CP and CP suspension in phosphate buffered saline (0.1 M) were poured into two dialysis bags (Millipore, molecular weight cutoff 12 kDa, USA) separately, and suspended into 100 mL phosphate buffered saline (0.1 M) with pH 7.4 at 37°C under constant stirring. The receptor compartment was sealed to avoid evaporation of the receptor phase. At predetermined time intervals, 1 mL of the released CP-PBS suspension outside of the dialysis bags were withdrawn and replaced with equal volume of the fresh receptor phase to complete the initial volume. Then CP absorbance was quantified by UV-Vis measurement and the released amount of CP was calculated according to the CP standard curve analysis.

#### Cell viability assay

MCf-7 cells (10^4^) were maintained in DMEM supplemented with 10% FBS and 1% penicillin/streptomycin on each well of 96-well culture dish and incubated at 37°C in a humidified atmosphere with 5% CO_2_ for 24 h. 100 µL of the Fe_3_O_4_/A/β-CD/CP composites at the various concentrations of 0.005, 0.043, 0.425, 4.25 and 42.5 µg/mL were applied to each well, followed by 24 h incubation. The next day 10 µL of MTT solution was added to each well and kept in the incubator conditions for 3 h. The absorbance of the samples was measured by ELISA reader (BioTek^®^ Elx 800) at 490 nm wavelength and the reference of 630 nm. Absorbance of non-treated cells (as control) was estimated as 100% viability and the treated cells were calculated according to that. The survival rate (%) was calculated according to the following equation ^20^:

% Survival rate = (OD in treatment group/OD in control group)×100.

#### Statistical analysis

Data were presented as mean ± SD while experiments were performed in triplicate. SPSS software version 15 for Windows (SPSS Inc., Chicago) was utilized for statistical analysis. The differences between the groups in the *in vitro* release profile of CPwere determined by employing *t* test analysis. Microsoft Office Excel 2010 was also used as appropriate statistical software.

## Results and Discussion

### Characterization of fabricated particles

#### FT-IR studies

**Figure 1:**
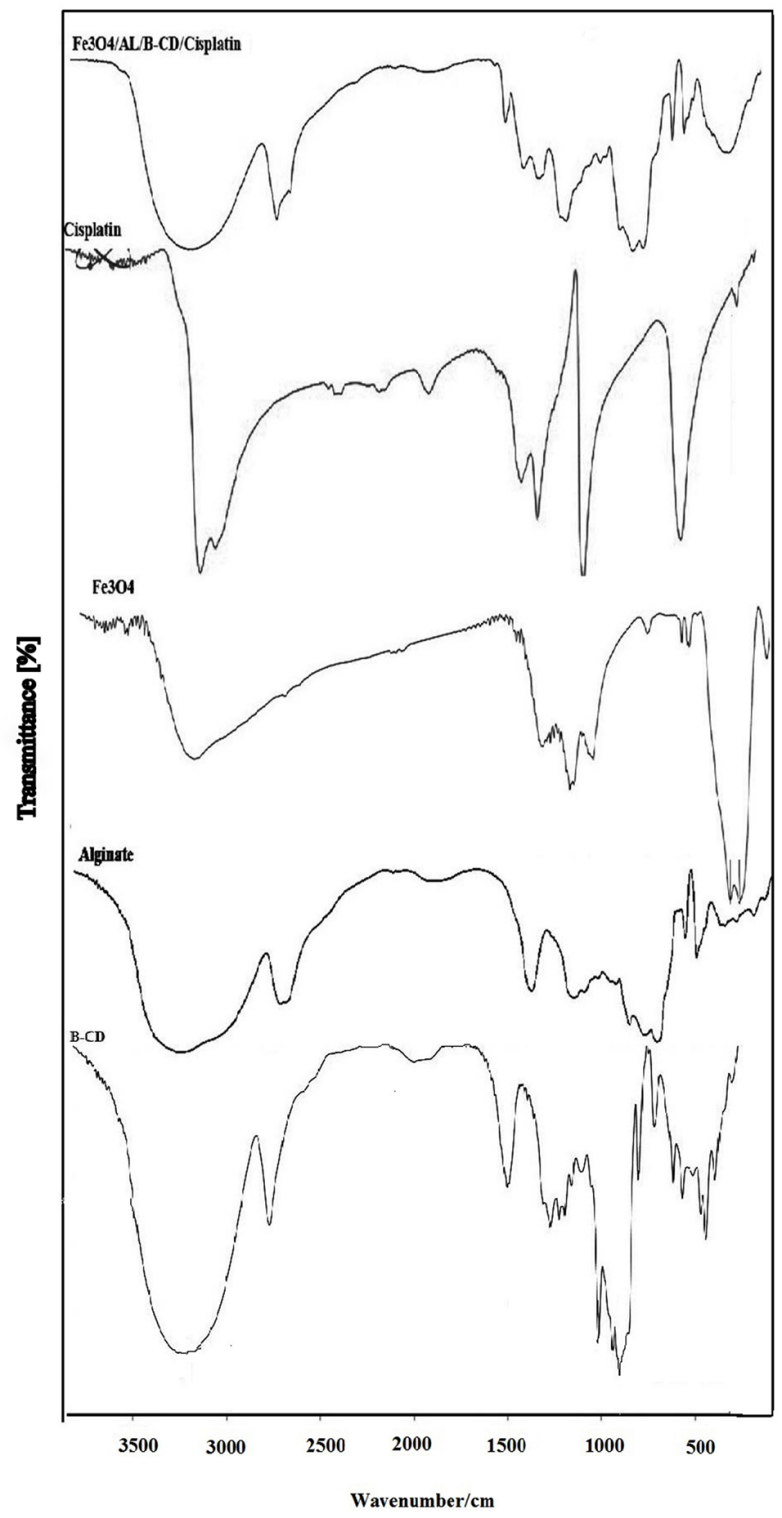
FT-IR spectra of cisplatin; β-CD; Sodium Alginate (SA); Fe_3_O_4_ and Fe_3_O_4_/SA/ β-CD/CP.

The FT-IR spectra ofCP, β-CD, sodium alginate, Fe_3_O_4_, Fe_3_O_4_/A/β/CD and Fe_3_O_4_/A/β-CD/CP are presented in Figure 1. Absorption peak presented at 1645.85 cm^-1^, belonged to the stretching vibration of the carbonyl group, this absorption also presents in the β-CD, which confirmed the presence of β-CD in the final product. The absorption band at 2924.42 cm^-1^, can be attributed to -CH stretching vibration in sodium alginate and β-CD. The band at 3283.56 cm^-1^, can be assigned to –NH_2_ stretching, and according to the peak at 3419.11 cm^-1^ in Fe_3_O_4_/A/ β-CD-CP that related to N-H stretching, and can be inferred that the absorption peak corroborated the attendance of CP is in the final composition. These results indicated to conformity mentioned compounds in pure form with Fe_3_O_4_/A/ β-CD/CP and confirmed the presence of these compounds in the final composite structure.

#### Particle size and zeta potential

**Figure 2:**
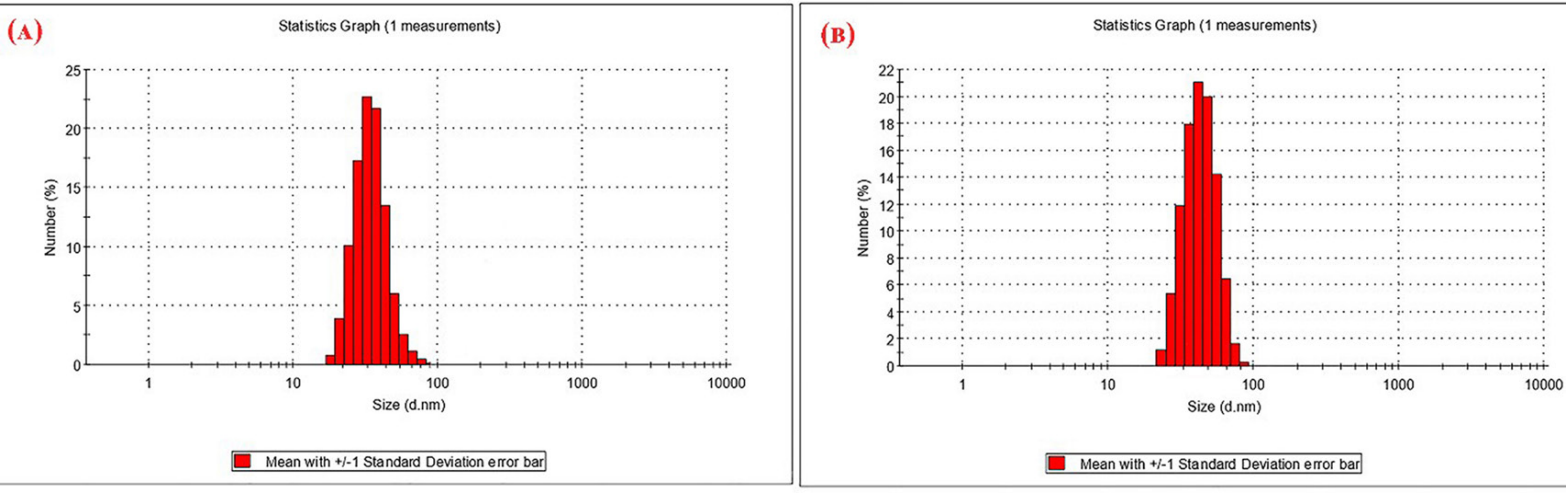
Particle size distribution of (A); Fe_3_O_4_/SA/β-CD and (B); Fe_3_O_4_/SA/ β-CD/CP.

Size distribution and zeta potential of Fe_3_O_4_/A/β-CD and Fe_3_O_4_/A/ β-CD/CP are shown in Figure 2. The average diameter of Fe_3_O_4_/A/β-CD was 57 nm while the mean size of Fe_3_O_4_/A/ β-CD/CP was 65 nm. Ζ-potential of the Fe_3_O_4_/A/β-CD was -16, while ζ-potential of the Fe_3_O_4_/A/ β-CD/CP was decreased to -10 by two peaks with sizes of -4 and -30 respectively, which could be represented an appropriate repulsion between particles and particle size stability.

#### FE-SEM studies

**Figure 3:**
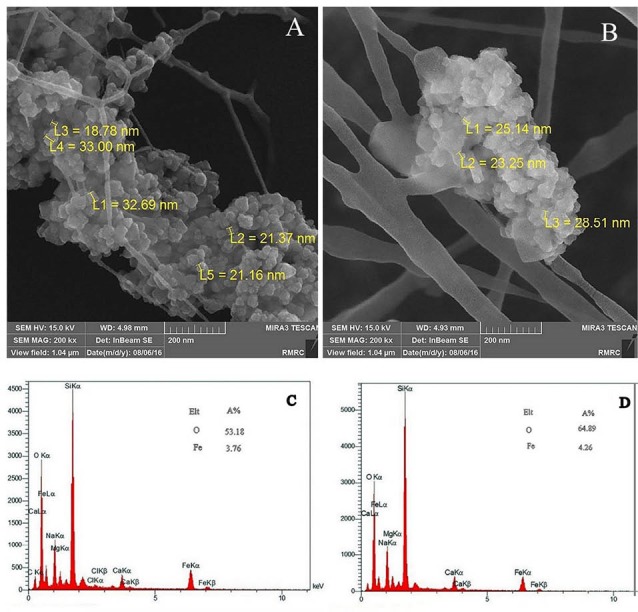
SEM micrographs of (A); Fe_3_O_4_/SA/β-CD and (B); Fe_3_O_4_/SA/ β-CD/CP and EDX analysis results of (C); Fe_3_O_4_/SA/β-CD and (D); Fe_3_O_4_/SA/ β-CD/CP respectively.

Results from FE-SEM analysis performed to examine the morphological aspect and size of Fe_3_O_4_/A/β-CD and Fe_3_O_4_/A/β-CD/CP and are depicted in Figure 3. According to the FE-SEM images, the mean particle size was about 50 nm and the shape of nanoparticles was elliptical to roughly spherical. Results from surface analysis of nanospheres indicated a lack of accumulation of nanoparticles that is one of the criteria for the proper functioning of the nanoparticles.^21,22^ EDX analyses performed on the surface of Fe_3_O_4_/A/β-CD and Fe_3_O_4_/A/β-CD/CP clearly indicated the presence of Ca, Fe, O, C, Cl and Na (Figure 3 C and D). The peaks of Fe indicated the excitation of electrons in this atom in atomic layers M and L and move them to the lower layers.

#### TEM studies

**Figure 4:**
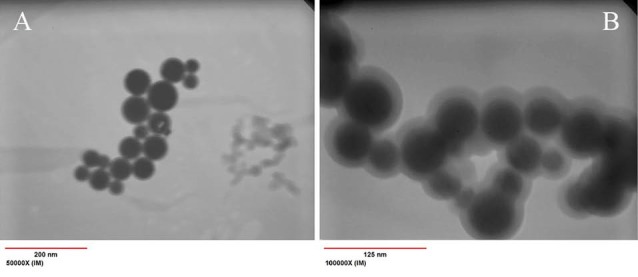
TEM micrographs of synthesized nanoparticles of (A); Fe_3_O_4_/SA/β-CD and (B); Fe_3_O_4_/SA/ β-CD/CP respectively.

The TEM images of Fe_3_O_4_/A/β-CD, and Fe_3_O_4_/A/β-CD/CP nanospheres are given in Figure 4. The average diameter of Fe_3_O_4_ nanoparticles was about 36 nm with a spherical shape. According to images, it could be seen that Fe_3_O_4_/A/β-CD and Fe_3_O_4_/A/β-CD/CP have the spherical morphology and the mean diameter of nanospheres was approximately 50 nm. As for being observed, the black crystalline core with a diameter about 36 nm is surrounded by gray layer with a diameter about 7 nm. It can assume that the preparation of magnetic supports was successful.

#### VSM studies

**Figure 5:**
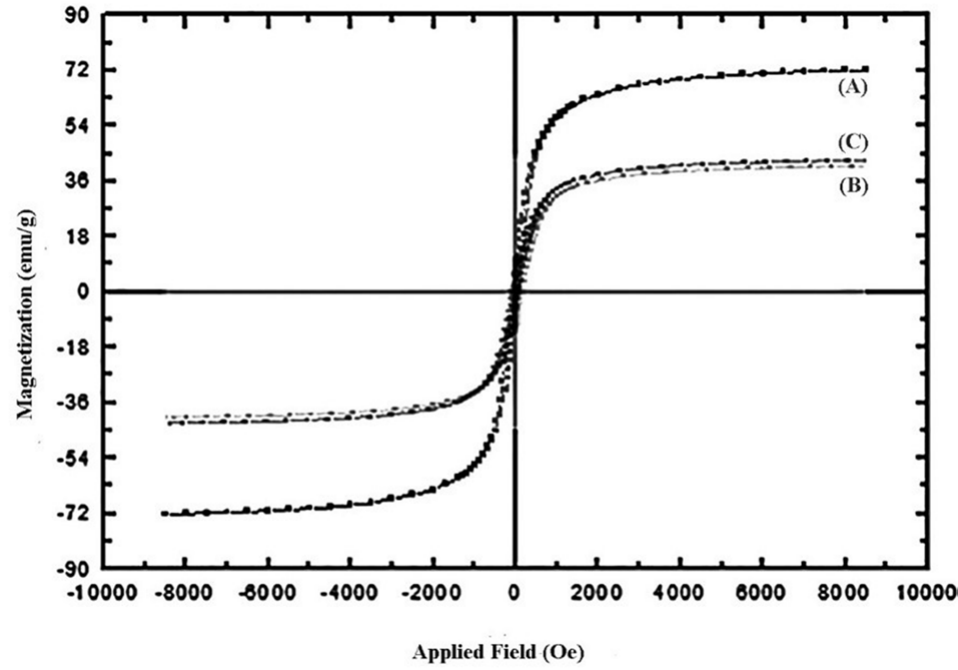
Magnetization hysteresis loop of (A); Fe_3_O_4_, (B); Fe_3_O_4_/SA/β-CD and (C); Fe_3_O_4_/SA/β-CD/CP respectively.

Magnetization curves (M-H loop) of Fe_3_O_4_, Fe_3_O_4_/A/β-CD, and Fe_3_O_4_/A/ β-CD/CP are illustrated in Figure 5. The saturation magnetization values of 72, 40, and 42 emus/g were measured for Fe_3_O_4_, Fe_3_O_4_/A/β-CD, and Fe_3_O_4_/A/β-CD/CP, respectively. As a result, these magnetic supports were used for carrying CP could be separated from the reaction medium rapidly and easily in an external magnetic field.^23^ Moreover, there was no hysteresis in the magnetization with both remanence and coercivity being zero, proving that these magnetic nanospheres were superparamagnetic.^24^ Therefore, these magnetic supports could respond to an applied magnetics field without any permanent magnetization and redisposed rapidly when the magnetic field disappeared. The prepared supports showed well super paramagnetic and better saturation magnetization.

#### STA studies

**Figure 6:**
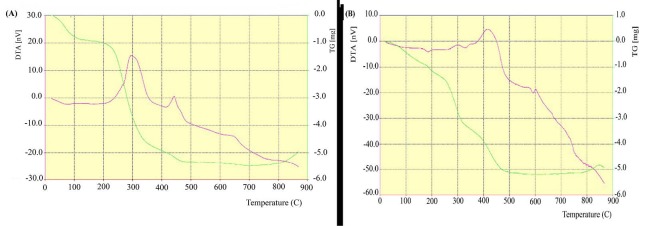
TG-DTA analysis of (A); Fe_3_O_4_/SA/β-CD and (B); Fe_3_O_4_/SA/ β-CD/CP respectively.

The TG-DTA analyses of Fe3O4/A/β-CD and Fe3O4/SA/β-CD/CP nanospheres are given in Figure 6. The TG curve was observed five stages of weight loss in a temperature range of (30-100°C), (100-200°C), (200-300°C), (300-400°C) and (400-470°C) respectively. The first stage of weight loss occurred in the range temperature of 30-100°C due to water evaporation surface of the nanospheres,^25^ and weight loss in the later stages was due to evaporation of useful water and organic compounds in the super paramagnetic nanospheres.^26,27^ That its weight loss was consistent with the presence of two exothermic peaks around 300°C and 430°C (Figure 6 A) for Fe_3_O_4_/A/β-CD and 300°C and 420°C (Figure 6B ) for Fe_3_O_4_/A/ β-CD/CP in the DTA curve, respectively.^28^

#### Drug loading measurements

Loading percentage of CP was the remaining amount on the system compared to the initial amount (50 µg). Data showed that about 85% of the drug was loaded on the Fe_3_O_4_/A/β-CD nanospheres. Thus it represents an excellent loading capabilities drug delivery system, which in turn is remarkable.^29,30^

#### Drug release measurement

**Figure 7:**
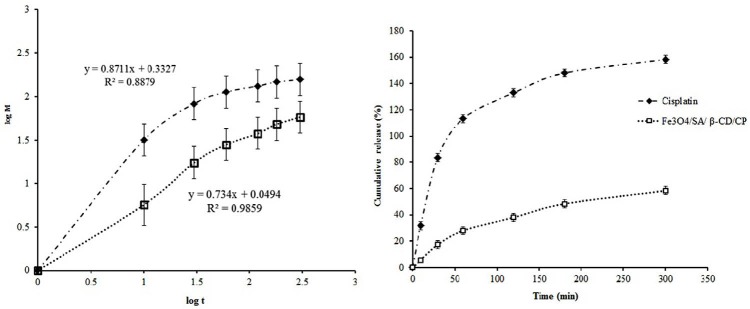
Cumulative release of CP from Fe_3_O_4_/SA/ β-CD composite after 300 min.

The release profiles of CP (blank) and Fe_3_O_4_/A/β-CD/CP suspensions after 300 min are shown in Figure 7. Results are expressed as released percent (mean ± SD) at different times. Clearly, blank exhibited very rapid releases in PBS solution; about 100% of drug over 1 h while Fe_3_O_4_/A/β-CD/CP composite showed a significant delay in the release, and only 30% of CP was released in PBS solution at the same period of time and very sustained release during 5 h. This is because of the drug deposited on the outer surface of the composites in the early hours and sustained release of the encapsulated drug in a long time. That confirms the appropriate capability and performance of the controlled drug delivery system that was designed.^31^ Drug nanoparticles showed the ability of full release of drug due to small size and larger surface area. The quantity of cumulated release of drug during 5 h was near 60%. The quantification of the R^2^ was used to determine the type of kinetic release. Zero-order, first-order, Peppas–Korsmeye, Higuchi, Weibull, and Hixon–Crowell are kinetic models that are used in *in vitro* release study.^32^ According to the R^2^ =0.9845 obtained from the *in vitro* release the Peppas–Korsmeyer model was the best fit with cisplatin release from Fe_3_O_4_/A/β-CD composites.

#### Cytotoxicity assay

**Figure 8:**
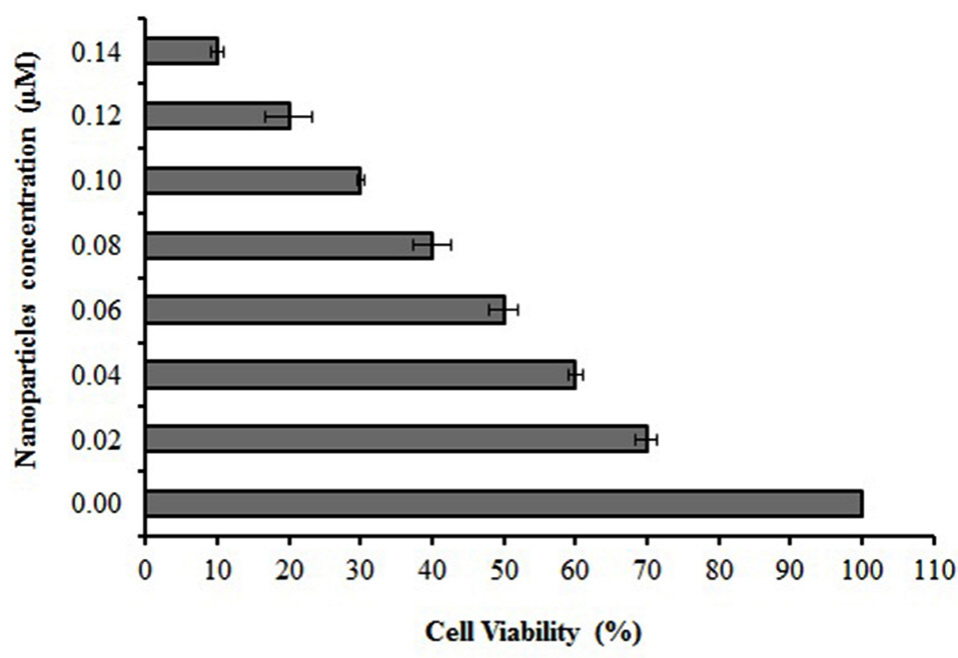
Dose-dependent inhibition properties of Fe_3_O_4_/SA/ β-CD/CP nanospheres after 24 h incubation, Data presented as mean ± standard deviation (n = 3).

The toxicity of various concentrations of encapsulated cisplatin in Fe_3_O_4_/A/β-CD composite was evaluated by using MTT tests to quantify cell viability (Figure 8). Results showed that as the concentration of encapsulated cisplatin rose from zero to 0.141 µM, the survival rate was decreased dose- dependently with an IC_50_ value of 0.06 µM, while the mean IC_50_ value of free cisplatin was obtained 5.75 and 8.6 µM.^33^ This means that Fe_3_O_4_/A/β-CD/CP composite could better decrease the cell progression during to the time of the incubation; this effect may be related to the drug release by delay.

## Conclusion

In general, it can be concluded that cisplatin was loaded sufficiently and efficiently on the Fe_3_O_4_/A/β-CD composites which could be released in a sustained, slow and controlled mannered. These drug delivery systems have a potential to response to the external magnetic stimuli which may be applied in the targeted cancer chemotherapy.

## Conflict of Interest

There is no conflict of interest.

## Ethical Issues

Not applicable.

## Acknowledgments

The work has been kindly supported by the Kerman University of Medical Sciences (KMU) of Iran.
